# Triglyceride Blisters in Lipid Bilayers: Implications for Lipid Droplet Biogenesis and the Mobile Lipid Signal in Cancer Cell Membranes

**DOI:** 10.1371/journal.pone.0012811

**Published:** 2010-09-22

**Authors:** Himanshu Khandelia, Lars Duelund, Kirsi I. Pakkanen, John H. Ipsen

**Affiliations:** MEMPHYS-Center for Biomembrane Physics, Department of Physics and Chemistry, University of Southern Denmark, Odense, Denmark; University of Oxford, United Kingdom

## Abstract

Triglycerides have a limited solubility, around 3%, in phosphatidylcholine lipid bilayers. Using millisecond-scale course grained molecular dynamics simulations, we show that the model lipid bilayer can accommodate a higher concentration of triolein (TO) than earlier anticipated, by sequestering triolein molecules to the bilayer center in the form of a disordered, isotropic, mobile neutral lipid aggregate, at least 17 nm in diameter, which forms spontaneously, and remains stable on at least the microsecond time scale. The results give credence to the hotly debated existence of mobile neutral lipid aggregates of unknown function present in malignant cells, and to the early biogenesis of lipid droplets accommodated between the two leaflets of the endoplasmic reticulum membrane. The TO aggregates give the bilayer a blister-like appearance, and will hinder the formation of multi-lamellar phases in model, and possibly living membranes. The blisters will result in anomalous membrane probe partitioning, which should be accounted for in the interpretation of probe-related measurements.

## Introduction

In this report, we investigate the biophysics of model membranes containing low concentrations of triglyceride molecules.

Triglycerides or triacylglycerols (TGLs) are neutral lipids where each of the glycerol hydroxyl groups is esterified by a fatty acid. TGLs are the major component of many natural oils such as olive oil. In mammals, TGLs are present mostly inside trafficking lipoprotein particles, which transport cholesterol, steryl esters, and TGLs between tissues [1] and in lipid droplets (LDs) [2]. LDs are also present in other eukaryots, and in some prokaryotic cells which synthesize TGLs for energy and carbon storage [3]. TGLs in lipoproteins and LDs have been the subject of much interest owing to the role of these particles in physiology [4], disease [5].

Besides lipoproteins and LDs, TGLs are also present in several biological membranes at varying concentrations. The lamellar bodies of lung surfactant extracts in mammals can contain between 0.5% to 1.8% w/w TGLs [6], [7]. Ocular lens lipids contain small amounts (µg TGLs/mg phospholipids) of TGLs. TGLs are also present in intestinal membrane extracts [8]. Lysosomes contain non-negligible amounts of TGLs, for example, in cultured hamster fibroblasts [9]. In rat hepatocytes, lysosomes contain nearly 3.7% TGLs [10]. Many proliferating or activated mammalian cells in particular, have a high concentration of TGLs in membranes. Cancer cells contain as high as 6.8% TGL fraction of total plasma membrane lipids [11]. Several malignant Chinese hamster ovary (CHO) cell lines contain 2.4–3.2% TGLs in their plasma membranes [12]. Human neutrophils contain as high as 5.2% and 6.8% TGLs in their plasma membranes before and after stimulation with lipopolysaccharides [13]. Activated macrophages [14], lymphocytes [15] and B cells [16] also contain high amounts of TGLs in their plasma membranes. In this report, we investigate the effect of low concentrations of TGLs, as found in a variety of cell types noted above, on the structure and dynamics of model membranes, with the objective of ultimately obtaining hints into the possible structural and functional role of TGLs in the plasma membrane of living systems. We have used triolein (TO) as our model TGL, and 1-Palmitoyl-2-oleoyl-*sn*-glycero-3-phosphocholine (POPC) as the model phospholipid.

There have been scattered reports investigating the biophysics and biochemistry of model TO-phospholipid mixtures. Several ^13^C-NMR studies have been used to determine the maximum solubility of TGLs in model membranes containing mainly PC lipids. The ^13^C spectrum of a TGL can distinguish between the carbonyl groups at different positions (α or β), and different hydration levels (near an interface, or in an oily droplet). Using ^13^C-NMR, the solubility of TO in lamellar structures and oriented as phospholipids has thus been documented to be within 3% both in small unilamellar vesicles (SUVs) [17], [18] and in multilamellar vesicles (MLVs) [19] of phosphatidylcholine lipids. Similar solubility limits were obtained when heterogeneous TGLs, where all three positions of glycerol are not esterified by the same fatty acid, were solubilized by SUVs [20]. In SUVs, the solubility is lowered to as low as 1% if cholesterol is present in a 1∶2 ratio with phospholipids and to 0.15% if the ratio is 1∶1 [21]. In all investigations, additional carbonyl peaks in the spectra were obtained when the TGL concentration was raised above 4%. These peaks were typical of TGLs in the “oil” phase. Multinuclear and magic-angle spinning NMR investigations of 0, 0.25,and 0.75 mole fraction TO in egg-phosphatidylcholine indicated that TO caused significant changes in the bilayer molecular organization, including an increase in chain fluidity [22]. TGLs, being shaped like inverted cones, can also promote the lamellar to inverted hexagonal phase transition in both PE membranes [23], [24].

Although only up to 2–3% TGLs can be detected as solubilized at the interface in model membranes, it is clear that proliferating cells (see above) can contain more than 6–7% TGLs. If model membranes can only accommodate 2–3% TGLs in the canonical bilayer conformation, and even less so if cholesterol is present [21], and more than 6–7% TGLs is indeed present in the membranes of cholesterol-containing living cells, *where does the excess TGL go*? One possible answer lies in the observation of a distinct group of ^1^H-NMR resonances from live cell NMR experiments, which originate from “mobile lipids” from tissue triglycerides [25]. It was proposed in the 1980s [26] that this signal partly originated from an excess triglyceride pool which is accommodated in the middle of the membrane as microdomains. There have been scattered reports of microscopic observations of such microdomains, although slightly bigger in size ∼60 nm [27], [28], [29]. Most of the ^1^H-NMR mobile lipid signal possibly comes from the cytoplasmic pool of TGLs, but based on microscopy studies and the sensitivity of the signal to paramagnetic gadolimium ions (which bind to membranes), the possibility of a membrane pool of TGLs cannot be ruled out [25].

In recent work, it was shown that centrifugation or overnight settling of TO-POPC mixtures led to a phase separation into a heavy phase pellet and a light phase, while the excess TO formed an oily later on top [30]. Although the composition of the light and heavy phase were similar, the two phases had remarkably different hydration and fluidity properties, some of which could be explained by a model which suggested that the glycerol backbone of TGLs could also be in the center of the lipid bilayer [30].

Prior simulations of TGL-containing systems have either focused on lipid emulsions [31], or on lipoproteins [32], [33]. In this study, we have investigated the conformations of TGLs in model POPC lipid bilayers at concentrations above and below the interfacial solubility limit of TGLs. We have used extensive course grained molecular dynamics simulations to access structural and dynamics details not easily accessible by analytical techniques. Besides obtaining new insights into the biophysics of TGL-containing membranes, our findings are relevant to the biogenesis of lipid droplets in the ER, and to the presence of mobile lipid domains in malignant or activated cells.

## Methods

Simulations were carried out using course-grained (CG) models of POPC-TO mixtures at a two concentrations, one slightly below (∼2.3%), and one above (∼5.2%) the interfacial solubility limit of TO. The complete list of simulations is shown in Table 1. CGMD simulations were carried out using the MARTINI force field for lipids [34]. Force field parameters for TO were adapted from existing parameters for DOPC, by replacing the phosphocholine moiety by an oleoyl chain. It was not required to introduce any new particle, bond or interaction types.

**Table 1:** List of completed simulations.

Name	# TO	# POPC	Time Simulated (µs)^1^	Flip-flop rate (# events/µs)
POPCCG	0	270	256	0
UNI2-1	6	256	192	1.15
UNI2-2	6	256	200	1.12
UNI5-1*	14	256	180	0.85
UNI5-2*	14	256	190	0.86
RAND5-1*	14	256	200	0.95
RAND5-2*	14	256	174	0.83
4X2	24	1024	124	1.26
4X5*	56	1024	24	0.31
4XMID5*	56	1024	27	0.32
9X5*	126	2304	29	-
16X5*	224	4096	64	-
SELF2×3	6	243	50+50+25	-
SELF5*×3	13	243	25+25+25	-

1 The time shown is actual simulated time multiplied by a factor of 4, to account for the faster sampling and thermal diffusion rates in course grained simulations.

List of completed simulations and flip-flop rates of TO. The 5.2% TO simulations are marked with an asterisk.

The initial coordinates of a POPC bilayer were obtained from a self-assembly simulation of 270 POPC in excess water, at a lipid∶water ratio of 1∶60. Since each water bead in MARTINI corresponds to four water molecules, the simulation contained 270 lipids and 4050 water beads. To construct the 5.2% TO-POPC mixture, 14 POPC molecules were replaced by TO molecules in two different ways. In one case, 7 random POPC molecules were picked from each leaflet and replaced by TO molecules. We will refer to this setup as RAND5. In the second case, uniformly spaced POPC molecules were selectively replaced by TO molecules. We will refer to this setup as UNI5. Two copies each of RAND5 and UNI5 setups were simulated with different initial velocity distributions, resulting in 4 simulations of the 5.2% TO systems. To construct the 2.3% TO systems, 8 TO molecules were removed from the UNI5 systems. We will refer to this setup as UNI2. Two copies of the UNI2 setup were simulated.

Simulations were also run for larger bilayer patches 19 nm×19 nm. For this, the UNI2 and UNI5 systems were replicated four times in the plane of the bilayer, resulting in bilayers containing 1024 POPC and 24 (2.3%) or 56 (5.2%) TO molecules. We will refer to these systems as 4X2 and 4X5 respectively. The final configuration from the UNI5 simulation was used to construct a bigger system using the same procedure, and a simulation of the bigger patch was implemented, we will refer to this setup as 4XMID5. Two very large simulations were implemented with 5.2% TO to test for finite size effects, and to check if the number and size of TO aggregates in the middle of membrane changed (see later). These systems will be referred to as 9X5 and 16X5. The bilayer patches were 28 nm×28 nm and 37 nm×37 nm, and the number of lipids was 2430 and 4230 respectively.

Finally, to confirm spontaneous partitioning of TO molecules into the lipid phase, self-assembly simulations of 2.3% and 5.2% TO containing randomly distributed POPC, TO and water molecules were implemented. Three copies of each concentration were simulated, and in each case TO partitioned into the self-assembled POPC bilayer within the first 100 nanoseconds. The data from the self-assembly simulations will not be discussed further, because the results are identical to those from the RAND5, UNI5 and UNI2 simulations.

The simulations were carried out using the GROMACS 4.0.4 package in the isobaric-isothermal (NPT) ensemble at 1 bar and 323 K, using the Berendsen [35] thermostat (relaxation time 0.3ps) and barostat (coupling constant 3.0 for smaller bilayer patches, and 5.0 for larger bilayer patches) with semi-isotropic pressure coupling. The Z-axis was parallel to the bilayer normal. A time step of 30 fs was used, coordinates were saved every 900 ps, and were subsequently processed to store snapshots every 13.5 ns to expedite analysis routines. Non-bonded interactions were cutoff at 12 Å.

Analysis of the simulations was carried out in the GROMACS suite of programs. VMD was used for molecular graphics [36]. The first 2 µs of the CG simulations were discarded as equilibration periods for calculation of ensemble-average properties.

## Results

### Distribution of Triolein in POPC

The structural properties of the bilayer, the density distribution of TO and POPC, and the flip flop rates of TO from the two copies each of the UNI5 and RAND5 were qualitatively and quantitatively similar, showing that the results discussed in the following sections are independent of the initial conformations of TO in the POPC bilayer. Furthermore, results from the larger 4X5 and 4XMID5 simulations were identical, indicating that the final conformations were independent of the initial state of the system. For clarity, results from only one out of the four CG simulations with 5.2% TO will be presented, unless otherwise indicated. Similarly, the results from only one of the two copies of UNI2 are presented.

**Figure 1:**
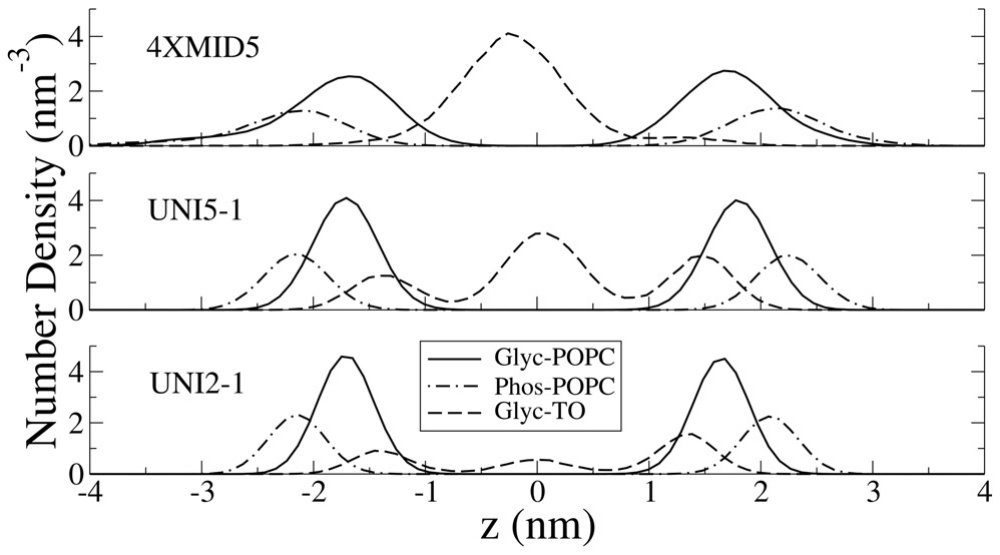
Density profiles of lipid moieties. Number density profiles of the glycerol backbone of TO (Glyc-TO), POPC phosphate groups (Phos-POPC) and POPC glycerol beads (Glyc-POPC). The concentration of TO at the interface is similar for the UNI2 (c) and UNI5 (b) simulations, but is very low in the 4XMID5 simulation (a), in which most of the TO partitions into the bilayer center.

**Figure 2:**
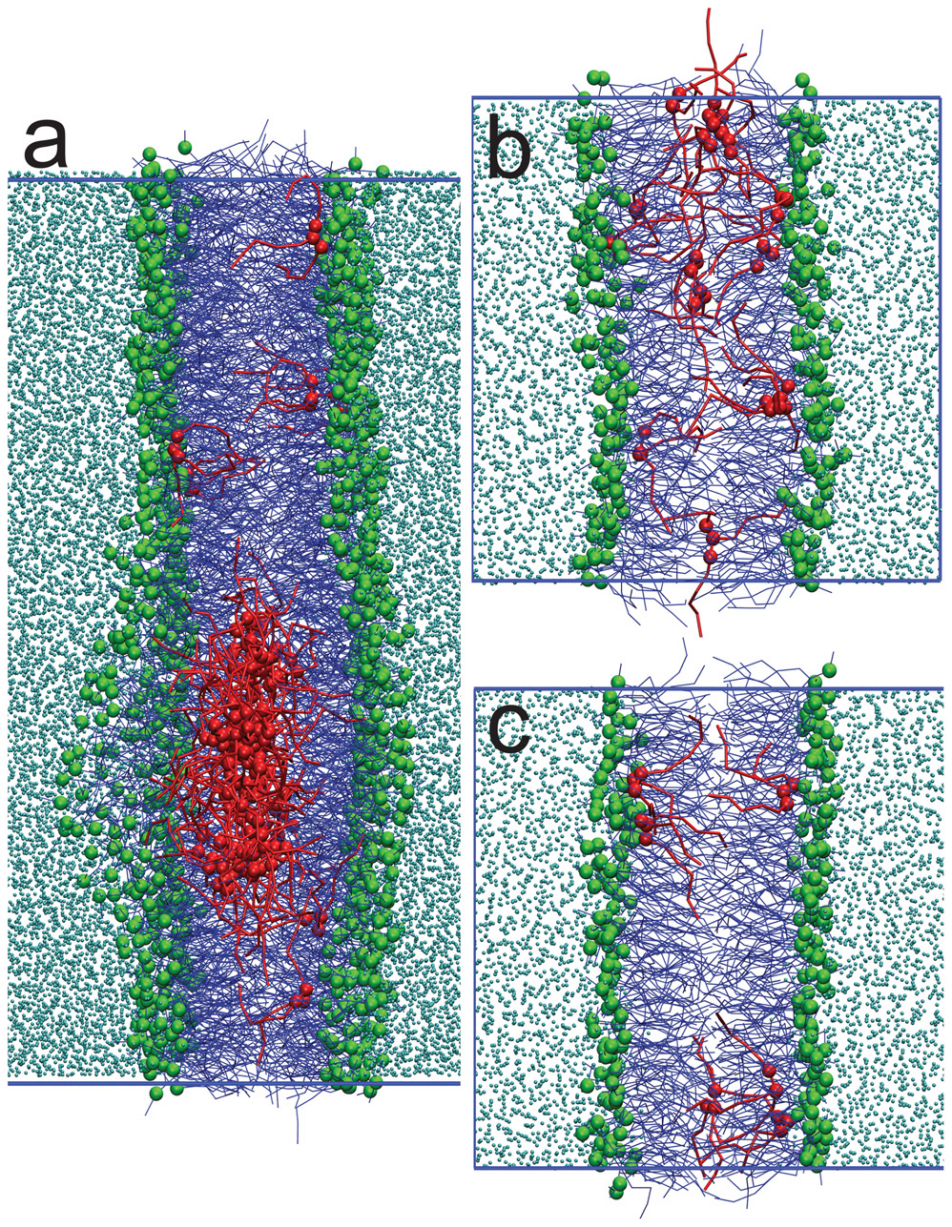
Simulation snapshots. Snapshots from the (a) 4XMID5 (b) UNI5 (c) UNI2 simulations. POPC tails are shown in blue, POPC phosphate beads are shown as green spheres, TO tails are shown in red, TO glycerol backbone reads are shown as red spheres, and water is depicted in cyan. The blue lines represent the central simulation cell boundaries.

The density distribution of the glycerol backbone of TO from the UNI2 (2.3% TO), UNI5 (5.2% TO) and 4X5 (5.2% TO, larger simulation size) simulations is shown in Fig. 1. Simulations snapshots from the three simulations are shown in Fig. 2. The density of TO components has been multiplied by a factor of 10 for clarity. Counter-intuitively, there is a significant density of the polar TO glycerol backbone residing in the hydrophobic center of the membrane in all three cases. There is a higher density of TO in the UNI5 simulation, compared to the UNI2 simulation. In the UNI5 simulation, some TO molecules partition into the bilayer center in the form of a TO aggregate, which is devoid of any specific arrangement of TO molecules. The number of TO molecules which remained at the interface were similar for the 2.3% and 5.2% simulations, indicating the limited interfacial solubility of TO in lipid bilayers. The excess lipids that could not be accommodated at the interface in the 5.2% simulations formed an aggregate in the middle of the membrane. However, as the system size increased, very little TO was left at the interface (Fig. 1a) in the 4XMID5 simulation. Thus, TO preferred to partition to the aggregate, rather than stay at the membrane interface in a sufficiently large system with a larger aggregate. There was very little TO at the interface in the 4X5, 4XMID5, 9X5 and 16X5 simulations, and the aggregate occupied an area almost half of that of the membrane patch (Fig. 2a). No aggregate was formed in the UNI2 simulations. We note that the non-zero density of TO at the bilayer center in the UNI2 system demonstrates that TO can partition into the bilayer center without an aggregate being present. The density profile of UNI2 is similar to that of the larger 4X2 system of the same concentration. The aggregate at the center of the membrane is biologically relevant. It is similar to triglyceride-rich mobile lipid domains previously detected using ^1^H-NMR spectra of cell suspensions that detect mobile lipid domains that carry a high amount of triglycerides [11], [26], [28]. The aggregate is also similar to a nascent lipid droplet observed in the ER membrane. We will return to this in the Discussion.

The density profiles in Fig. 1 are not symmetric about the bilayer center. In particular, there are more TO molecules partitioning in the upper leaflet (z>0) compared to the lower leaflet, despite the high rate of TO flip-flop. The reason for this apparent anomaly is that the number of POPC lipids in the two leaflets of the bilayer was not equal in the initial lipid self-assembled POPC bilayer, which was used to subsequently construct all the TO-POPC simulation models. The number of POPC molecules in the upper leaflet was lower than in the lower leaflet, thus resulting in a larger number of TO molecules ultimately diffusing to the upper leaflet to keep the molecular density equal in both leaflets. We implemented two additional simulations (with 2.3% and 5.2% TO) where the initial number of POPC molecules was equal in both leaflets, and in that case, TO was distributed equally in both leaflets (Figure S1).

The flip-flop rate of TO molecules in all CG simulations is reported in the last column of Table 1. Based on the density distribution of the TO glycerol backbone in each simulation, the bilayer was divided into three regions, two interfacial and one in the middle of the membrane. A flip-flop event was recorded every time the center of mass of the glycerol backbone of a TO molecule translocated from one interfacial region to the other. The flip flop rate of TO was remarkably high: ranging from 0.3 to 1.2 per µs of sampling time, depending on the concentration of TO, and size of the simulation. A flip-flop rate of ∼1 per µs sampling time would go undetected in shorter all-atom MD simulations. The flip-flop rate in the RAND5 and UNI5 simulations was higher because of the formation of the TO aggregate which lowers the free energy barrier for the translocation of the polar TO glycerol backbone across the membrane. The size of the aggregate increases almost proportionally in the larger 4X5 and 4XMID5 simulations, and the flip-flop rates simultaneously increase by nearly a factor of three, apparently because of a further decrease in the free energy barrier, as the aggregate occupies nearly half of the surface area of the bilayer patch. The aggregates formed in the 5.2% simulations are highly dynamic in nature, and TO molecules exchange often between the interface and the aggregate. No flip-flop event was observed for any POPC molecule in any simulation in agreement with time-resolved small angle neutron scattering experiments on large unilamellar vesicles, which indicate very slow rate of POPC flip-flop [37]. We also implemented about 1 µs simulations using higher resolution united-atom models for 0% and 5% TO using the Berger force field for lipids. In these simulations, no TO was seen at the bilayer center, and none of the TO molecules flip-flopped due to the short time scales.

### Molecular Conformations of Triolein Molecules

**Figure 3:**
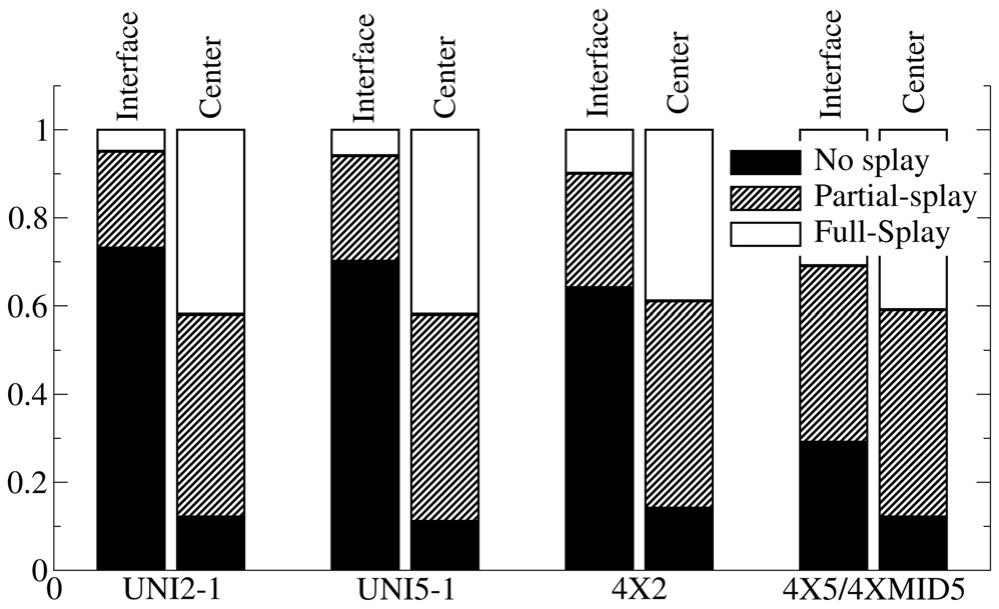
Splay fractions of TO molecules. The fractions of TO splayed tails in the CG simulations. For definitions of splaying, please refer to the text. The tails of a TO molecule are more splayed when it is near the bilayer center, suggesting an isotropic environment in the TO aggregate.

The three acyl chains of triolein molecules can splay with respect to each other, leading to various molecular conformations. Previous MD simulations of pure TO systems in crystalline or amorphous states showed that the molecules could adopt several conformations including the tuning fork (sn1 and sn3 tail parallel, and sn2 antiparallel), the chair (sn1 and sn2, or sn2 and sn3 parallel, and the other tail antiparallel), the trident (all tails parallel) or the liquid (chains pointing in random directions) [32]. Inside lipid bilayers, the same richness of conformations will not be replicated, because when at the interface, the packing of the tails of a TO molecule would be constrained to be parallel to the bilayer normal. However, when a TO molecule is at the center of the bilayer, its acyl chains can splay. We have divided TO conformations into three types depending on the angle between adjoining pairs of tails. *θ_1-2_* was defined as the angle between sn1 and sn2 tails, and *θ_2-3_* was defined as the angle between the sn2 and sn3 tails. The line joining the glycerol bead to the last acyl tail bead described each tail vector. We classified TO conformations as fully splayed, partly splayed or not splayed depending on *θ_1-2_* and *θ_2-3_*:
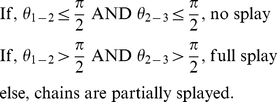
The overall fraction of the unsplayed, fully splayed, and partially splayed TO molecules is shown in Fig. 3. The extent of splaying was correlated to the position of a TO polar glycerol backbone inside the membrane. As expected, the acyl tails were more splayed in the center of the bilayer in all simulations. Contrary to intuition, however, the chains are partially splayed more than 20% of the time, and fully splayed 5% of the time when the polar backbone of the TO molecules resides at the interface. The fraction of fully splayed and partially splayed TO molecules is almost identical in all systems in the middle of the membrane. In the 5.2% TO systems, the overall splayed fractions are higher because the TO molecules spend more time in the middle of the membrane. For reference, the angle between the POPC acyl chains was greater than 90 degrees less than 4.5% of the time in all CG simulations (data not shown). The high proportion of splayed tails at the bilayer center suggests an isotropic TO aggregate.

### Hydration Levels of Glycerol Beads

**Figure 4:**
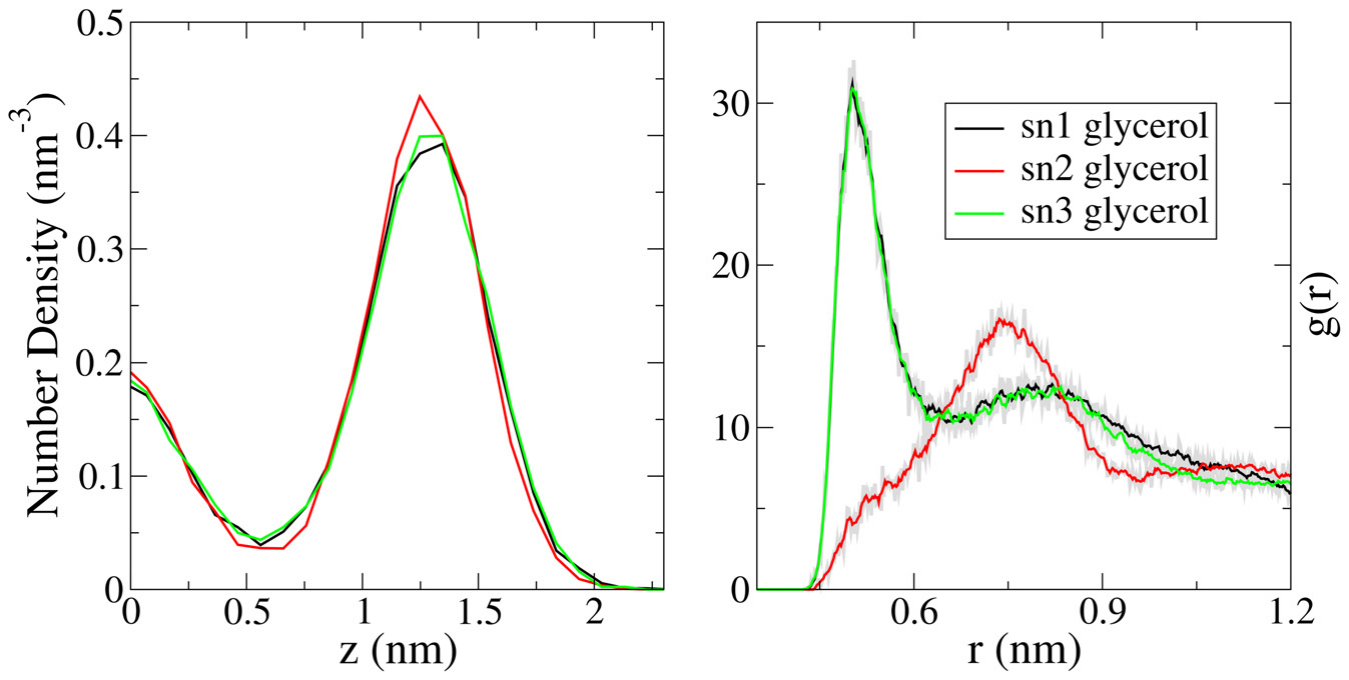
Hydration of carbonyl beads, agreement with NMR. (a) Density distribution of the two α and one β glycerol backbone beads of TO. For brevity, only one monolayer is shown. The β bead is located deeper in the bilayer than the two α beads. (b) radial distribution function of water around the beads. The β glycerol bead is clearly less hydrated than both the α beads, in very good agreement with ^13^C-NMR [17].

In the ^13^C-NMR spectra of TO in egg-PC unilamellar liposomes, it was possible to distinguish the two peaks arising from the two α-carbonyls and the β-carbonyl group [17]. The higher carbonyl resonance frequency β-carbonyl indicated that it was less hydrogen-bonded to water, and therefore sat deeper in the lipid-water interface [17]. In Fig. 4a, we have shown the density profiles for the three glycerol beads from the CG simulations along the bilayer normal. For clarity, only data from the small 2.3% TO system is reported. The sn2 (or β) carbonyl group is slightly deeper in the bilayer than the other two (sn1 and sn3) carbonyl beads. The hydration of the beads was calculated from the radial distribution function of water beads around each glycerol backbone bead (Fig. 4b). The β or sn2 bead is the least hydrated part of the glycerol backbone, in good agreement with NMR data.

The structural properties of the bilayer (area per lipid, thickness, order parameters) are presented in the supporting information. The areas and thicknesses are tabulated in Figure S2, and the order parameters are presented as Text S1.

## Discussion

**Figure 5:**
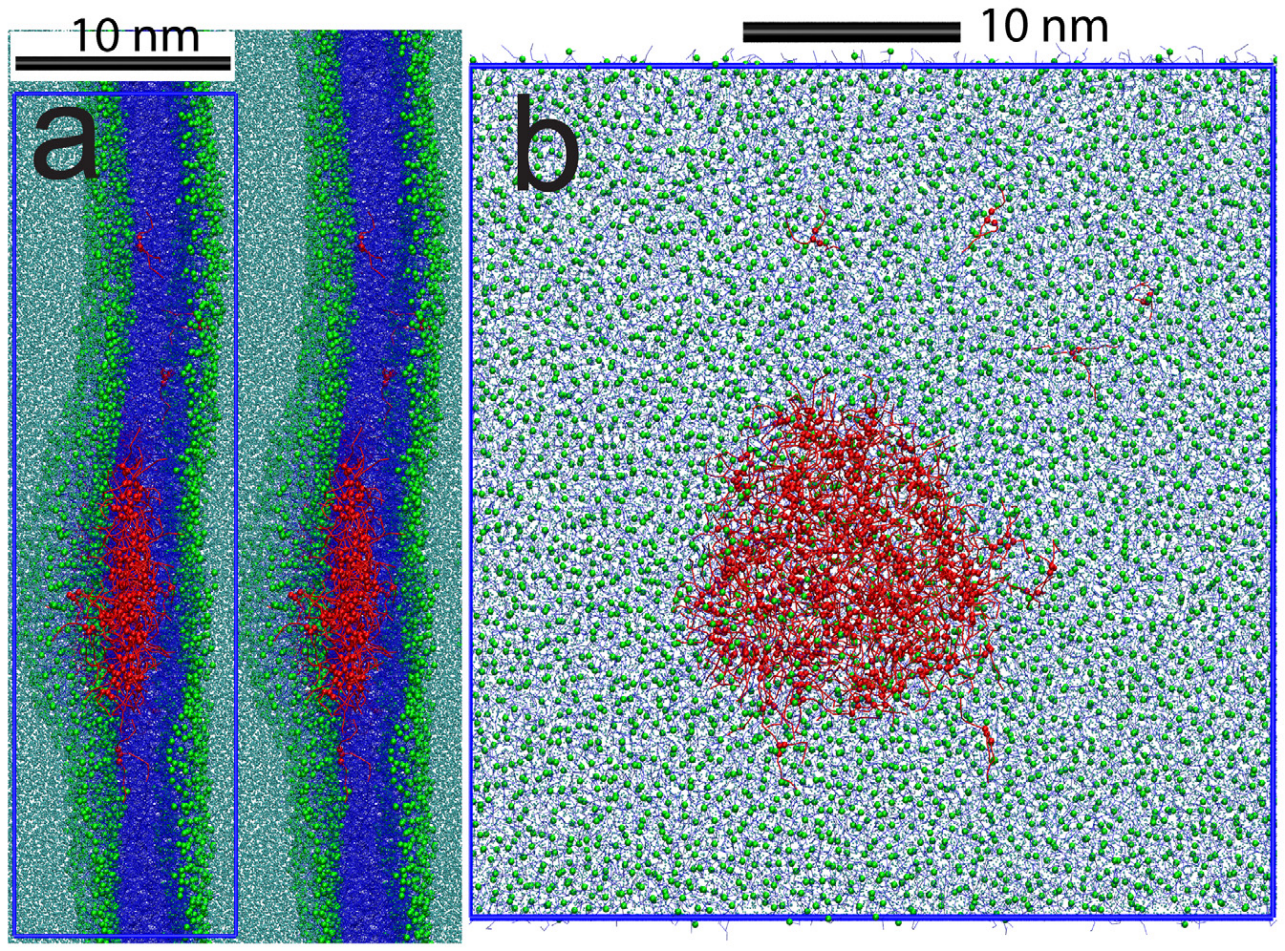
Simulation snapshots of 37×37 nm patch. Simulation snapshot from the largest system 16X5 simulated, containing a 37×37 nm bilayer patch. (a) Side view, where two periodic images in along the bilayer normal have been shown. (b) Top view, only the central simulation cell is shown. The blue lines represent the central simulation cell boundaries. The color-coding is similar to Fig. 2. The aggregate at the center is ∼17 nm in diameter.

Using extensive CG simulations summing to more than 1.8 milliseconds, we provide evidence for the presence of a large TO aggregate at the bilayer center at a TO concentration higher than the interfacial solubility of TO. As the simulation system size increases, the size of the aggregate also increases proportionally. In the largest simulations we implemented (1400 nm^2^ patch), the aggregate sucked in most of the TO molecules from the interface to the membrane center (Fig. 5). The aggregate was disc shaped and about 17 nm in diameter. In the 16X5 and 9X5 simulations, multiple aggregates were initially formed in the membrane, which subsequently coalesced together to form the larger aggregate. It is not clear if the size of the aggregate will continue to increase as increasingly larger bilayer patches are simulated, but finite-size effects are important in the current system. For the smaller UNI5 and RAND5 simulations, a significant density of TO was detected at the bilayer interface. However, as the simulation size was increased by 4X, 9X and 16X, most of the TO partitioned into the aggregate at the bilayer center.

When, and at what critical size (if any) will the TO aggregates leave the bilayer and phase separate? Triglyceride is insoluble in water and phase separates with a large interfacial tension *γ_O/W_* = 35–50 mN/m. When amphiphiles, e.g. surfactants or lipids, are introduced into the solution the interfacial tension is strongly diminished leading to micro-phase separation of water and oil. This is caused by the enrichment of the oil-water interface by a monolayer of amphiphiles. The interfacial free energy (*F_i_*) of such a self-assembled amphiphilic interface is must be modified to include bending elastic energy [38]

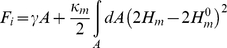
Here *H_m_* is the mean curvature of the amphiphilic monolayer and 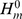 is the spontaneous curvature of the interface. *κ_m_* is the bending rigidity [39], and *A* is the interfacial area. For insoluble amphiphiles (like lipids such as POPC) the interfacial tension *γ* is vanishing and the above equation leads to a characteristic droplet size with the radius 1/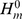. For a spherically shaped self-assembled monolayer the spontaneous curvature can be estimated from the critical packing parameter *P = v/al* [40], [41]: 
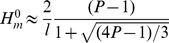


Here *l* is the molecular length, *v* is the molecular volume and *a* is the cross-sectional area of the headgroup. For POPC, 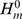 is about 13.9 nm. This is the classical droplet micro-emulsion picture of the ternary mixture of oil, water and lipids [42]. The present work has demonstrated that another scenario is possible in at low oil concentrations. The lipids form bilayer structures, which up to some solubility limit c* (3% for TO in POPC) allow the incorporation of the triglyceride into the bilayer. When [TO]<c^*^, TO partitions between a configuration in the center of the bilayer and an interfacial configuration with a partition coefficient of about ∼0.27 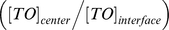. When [TO]>c^*^, the excess TO phase separates within the membrane structure, to form a pure oil phase in the center of the bilayer as seen in our simulations. Similar to the above description of the droplet micro-emulsion, triglyceride blisters form with a lipid monolayer at the oil-water interface, with the same preferred curvature 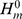. However, the overall size of the blisters may be smaller, due to boundary effects between the bilayer and the blister. The contribution of such boundary effect on the shape and stability of such blisters has recently been described by Zanghellini et al. [43] in a phenomenological model. It is also possible that blisters within lipid bilayers can be formed by other hydrocarbons as well, and such a possibility is worth investigating in the future.

What is the biological relevance of the TO aggregates? Most natural bilayer membranes do not contain a very high percentage of TGLs. However, the plasma membranes of certain activated or proliferating cell types, including cancer cells, macrophages, B and T cells, can contain more than 5% TGLs, although the function or implication of the presence of such a high amount of TGLs is unclear [27]. Since the interfacial solubility of TGLs is less than 5%, the conformational spectrum of TGLs in the plasma membranes of such cells is debatable. Suggestions based on ^1^H-NMR of mobile lipids that TGLs could form 20–30 nm sized microdomains at the bilayer center [26] did not gather support owing to lack of physical evidence of such domains, which have only appeared sporadically [28], [29]. A distinct mobile lipid ^1^H-NMR signal arising from 60 nm intra-membrane particles could be observed in TEM images freeze-fractured cells [28]. The particles were distinct from calveolae, and the signal was therefore attributed to a mobile lipid particle consisting of TGLs and steryl esters [28]. However, it was subsequently debated whether or not such a ^1^H-NMR signal could detect particles of this size at all, albeit for a different cell line [27]. Based on ^1^H-NMR experiments, Mountford and Wright had proposed a membrane model where lipid particles could be accommodated in plasma membranes. Mountford and Wright [26] argued that most of the ^1^H-NMR signal originated from the membrane, but this has been disputed since [25]. Simulations reported in this work provide introductory molecular evidence of structures where TO can indeed be present inside bilayer membranes in the freely tumbling form of “mobile lipids”. The size of the aggregates observed in our simulations is not as large as 60 nm, simply because we could not model systems large enough for such an aggregate to form. Thus, simulations lend support to original model of Mountford and Wright, with the notable exception that we are hesitant to call our aggregates “domains” because their maximum size (so far) is limited to 17 nm. Moreover, we find that the aggregates are highly dynamic in nature, and TO molecules can exchange between the aggregate and the interface at the time scale of microseconds.

Small TO droplets can also be found in the cytoplasm of several types of mammalian and yeast cells, in the form of lipid droplets. Lipid droplets (LDs), earlier considered merely energy depots, have been recently acknowledged as cellular organelles. LDs have a core formed by neutral lipids, which e.g. in adipocytes are for the most part TGLs. The core is lined by a lipid monolayer, which is derived from the mother membrane of the ER. However, the lipid composition of the LD monolayer differs from that of the ER membrane. The monolayer is abundant in phosphatidylcholine, phosphatidylethanolamine and phosphatidylinositol like the ER, but contains more lysolipids and free cholesterol and less sphingomyelin and phosphatidic acid than the mother membrane [44], [45]. Reasons behind enrichment and depletion of certain lipid species in the LD monolayer are not known. One of the proposed theories is that the biogenesis of LDs on the ER membrane occurs in special areas, or domains. These would, according to the assumption, contain a particular lipid composition as well as the enzyme machinery required for LD formation [46], [47]. Different theories on the initial formation of LDs have been put forward. Two of the proposed models are based on neutral lipids accumulating between the two leaflets of the ER membrane [48], [49], [50]. Another markedly different model suggests LD formation to occur through cellular vesicle-forming processes [51]. Our present results provide molecular level support for the models in which the LD initially grows inside the ER membrane leaflets as an oily aggregate [48], [49], [50]. The centers of masses of TO and POPC are not coincident in the 16X5 simulation, which is a simulation artifact resulting from an asymmetric distribution of phospholipids in the two leaflets (see Results section). However, the asymmetry shows that slightly lower density of phospholipids in one leaflet can drive the diffusion of the aggregate in one direction. Phospholipid asymmetry might facilitate processes where the LD is detached from the mother membrane from one side, pointing into the direction of one of the existing theories, in which the droplet inside the membrane is pinched off to the cytoplasm [48], [49]. Indeed, blisters or protuberances such as the ones depicted in Fig. 5 have been observed in TEM images of the electron microscopy images of the ER membrane of plant cells [49].

The surface of the two lipid monolayers surrounding the aggregate is highly curved (Fig. 5). Therefore, at sufficiently high concentration (5.2%), TGLs can induce high local curvature in lipid bilayers, and thus might directly influence several membrane-associated cellular processes [52]. From the more biophysical perspective, the presence of such aggregates alters the planar bilayer geometry, and leads to “blister” like formations on the bilayer. The presence of such blisters can possibly hinder formation of multi-lamellar structures, because adjacent lamellae may get too close, and fuse [53]. Formation of multivesicular lipid particles, which consist of non-concentric unilamellar vesicles of different sizes enclosed inside a larger vesicle, also requires the presence of neutral lipids like TGLs [54]. It is possible that the role of TGLs in such particles is to hinder the formation of multilamellar structures.

The lipid bilayer's permeability to molecules such as TGLs and possibly drugs can be increased by the formation of the TO aggregate. This is demonstrated by the higher flip-flop rate of TO in the 5.2% simulations, in which the aggregate is present. Furthermore, one or two beads of water could be seen in the aggregate in the 16X5 simulations. Neutral lipid probes used for fluorescence, such as the environment-sensitive probe Laurdan, or the Electron Paramagnetic Resonance (EPR) probe methyl-5-doxylstearic acid spin-label (Me-5-DSA); which are normally presumed to reside at the membrane interface, can be similarly induced to partition into the bilayer center by a high concentration of TGLs. If the probes do partition into the disordered, isotropic TO aggregate at the bilayer center, they will report a more fluid and isotropic environment than at the bilayer interface. The lower order and high hydration properties in 5% TO-incorporated POPC membranes, as detected by Laurdan General Polarization and EPR experiments [30], is readily explained by such anomalous probe partitioning. When a lower amount of TO is incorporated in the bilayer, both Laurdan and the EPR probes detect an ordered environment typical of a bilayer interface [30].

Neutral lipids such as diacylglycerols can flip-flop at much higher rates compared to phospholipids in lipid bilayers [55], [56]. Being more hydrophobic, TGLs will flip-flop at even higher rates as seen in the simulations. 2.3% TGL in POPC does not have a significant impact on structure or dynamics of the membrane. The interfacial fraction of TGLs in all our simulations have the β-glycerol bead less hydrated than the two α-glycerol beads, in very good agreement with ^13^C-NMR data.

In summary, the simulations reported here provide evidence for the formation of stable, disordered, isotropic, mobile triolein aggregates at the center of a planar lipid bilayer, at concentrations ∼5% TO. In biological systems, similar aggregates, but of a larger size, can be observed in activated or malignant mammalian cells which contain high amounts of TGLs, and in ER membranes where TGLs are synthesized. Our work therefore provides credence to the existence of the “mobile lipid domain” rich in TGLs, residing in activated cells, and to the theory that lipid droplets are indeed synthesized in the ER. Such aggregates will give the membrane a blister-like appearance, and destabilize the formation of multilamellar phases in model membranes, and perhaps even in living cell organelles like lysosomes. The partitioning free energy of neutral lipid molecules and lipid probes will be lowered by the aggregate, and might result in anomalous probe partitioning into the bilayer center, which should be taken into account when interpreting fluorescence, EPR, or other probe-related measurements. The experimental evidence of TGL aggregates such as those seen in our simulations comes mainly from TEM images of freeze fractured activated cells, from the ^1^H-NMR mobile lipid signal obtained from live cells, from EM images of the ER membrane, and the isotropic environment detected by EPR and Laurdan GP in POPC membranes containing 5% TO [30]. If TGL-like oils can indeed be accommodated at the bilayer center, as shown by our data, then the solubility levels of TGLs in planar lipid bilayers are higher than earlier reported.

The next step in the analysis of TO-POPC systems will be simulations of much larger membrane patches, where it will be possible to predict the maximum size of the TO aggregates in membranes, and include steryl esters in the mixture. Introduction of cholesterol, which is present in high concentration in mammalian plasma membranes, is known to influence TGL absorption into membranes, and it will be of interest to investigate the molecular and phase behavior of TGL-POPC-cholesterol ternary mixtures.

## Supporting Information

Figure S1 (0.43 MB DOC)

Figure S2 (0.78 MB DOC)

Text S1 (0.05 MB DOC)
