# Supplementary material for: Triglyceride Blisters in Lipid Bilayers: Implications for Lipid Droplet Biogenesis and the Mobile Lipid Signal in Cancer Cell Membranes
Source: PLoS One. 2010 Sep 22;5(9):e12811. doi: 10.1371/journal.pone.0012811 (PMC2943900; doi:10.1371/journal.pone.0012811)
Supplement: Figure S1 — (0.43 MB DOC) [file pone.0012811.s001.doc]

**Supporting Figure S1. TO Distributes Uniformly in the two Bilayer Leaflets**

In the simulations reported in the main paper, the number of POPC lipids in the two leaflets was not equal, resulting in an asymmetric distribution of TO molecules in the two leaflets. Simulations UNI5 and UNI2 were repeated with the number of POPC lipids equal in the two leaflets in the initial structure. These simulations are called EQUAL5 and EQUAL2 respectively. In these simulations, TO molecules distribute uniformly in the two leaflets, as shown by the figure below.

**
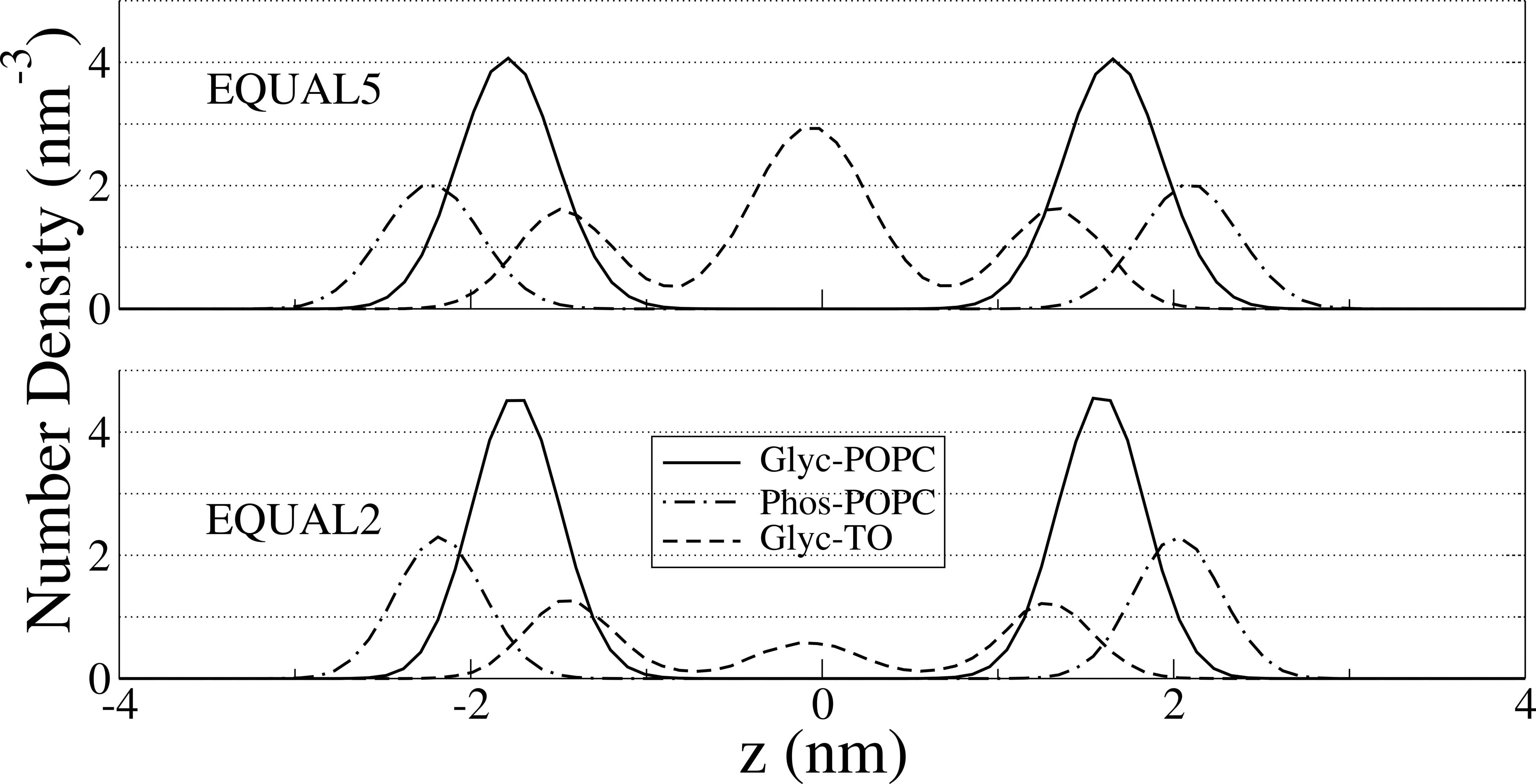
**

**Figure S1**: Number density profiles of the glycerol backbone of TO (Glyc-TO), POPC phosphate groups (Phos-POPC) and POPC glycerol beads (Glyc-POPC) in simulations with equal number of POPC molecules in each leaflet.
