# Supplementary material for: Triglyceride Blisters in Lipid Bilayers: Implications for Lipid Droplet Biogenesis and the Mobile Lipid Signal in Cancer Cell Membranes
Source: PLoS One. 2010 Sep 22;5(9):e12811. doi: 10.1371/journal.pone.0012811 (PMC2943900; doi:10.1371/journal.pone.0012811)
Supplement: Figure S2 — (0.78 MB DOC) [file pone.0012811.s002.doc]

**SUPPORTING FIGURE S2. Projected Area per Lipid and Bilayer Thickness**

The area per lipid (A_L_) for pure POPC was 67.16 ± 0.0005 Å^2^, which compares favorably with previously reported values in X-ray experiments (Kucerka, Tristram-Nagle et al. 2005). Introduction of 2.3% TO resulted in a negligible 0.58% increase in A_L_, while 5.2% TO in the RAND5 and UNI5 systems caused a 0.62% decrease in A_L_, because the bilayer thickened due the partitioning of TO at the bilayer center. The effect was enhanced in the larger 4X5 and 4XMID5 systems, because the size of the aggregate increased, resulting in a higher average thickness, and a 5% lower projected area per lipid. The area per lipid and thickness are presented in Supplmentary Fig. S2


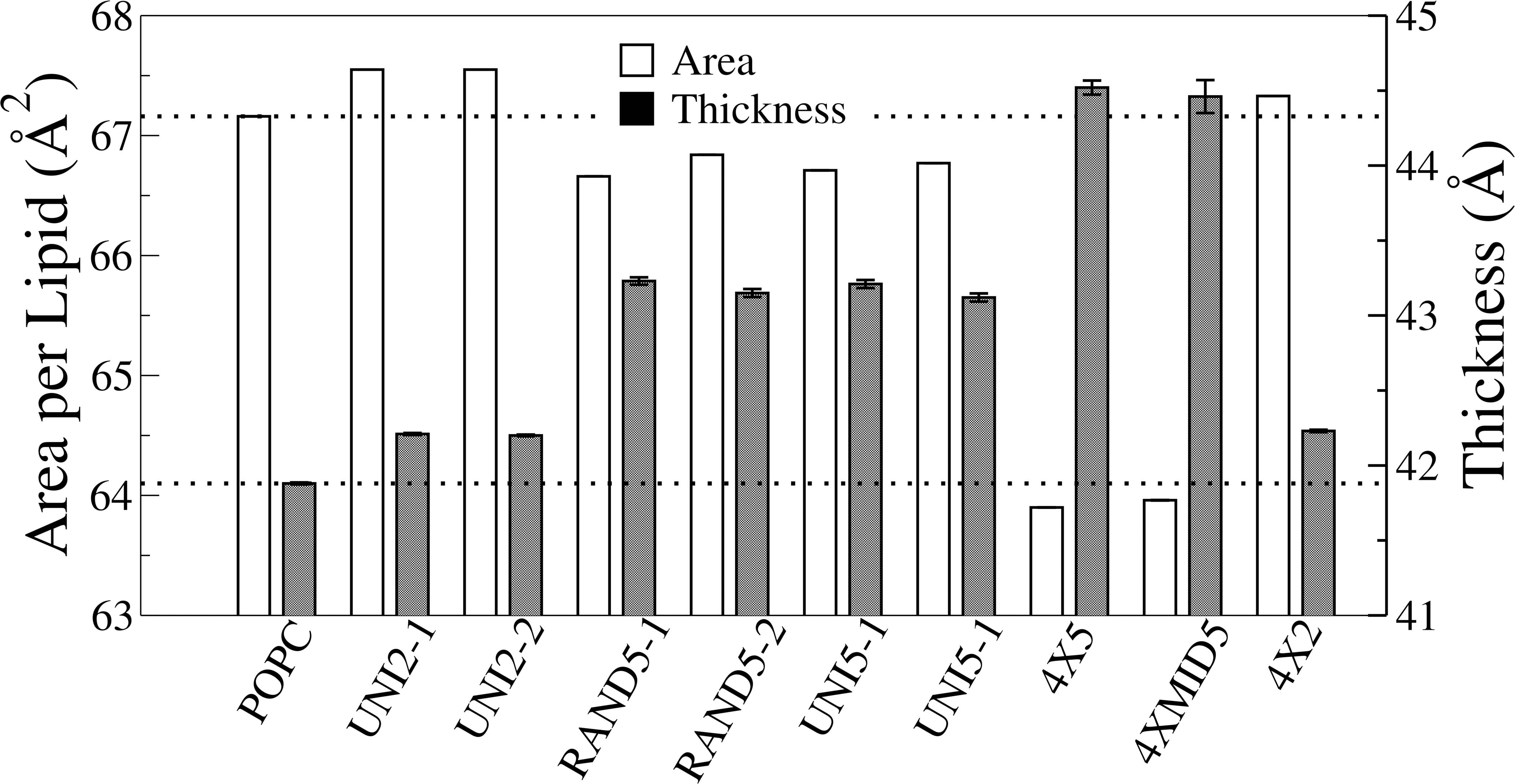


**Figure S2**: Projected area per lipid and the P-to-P thickness of the bilayers in the CG simulations. The area was calculated by dividing the box size by the number of lipids, and the thickness was calculated as the average distance between the center of mass of the phosphate beads of POPC in the two leaflets.
