# Supplementary material for: Triglyceride Blisters in Lipid Bilayers: Implications for Lipid Droplet Biogenesis and the Mobile Lipid Signal in Cancer Cell Membranes
Source: PLoS One. 2010 Sep 22;5(9):e12811. doi: 10.1371/journal.pone.0012811 (PMC2943900; doi:10.1371/journal.pone.0012811)
Supplement: Text S1 — (0.05 MB DOC) [file pone.0012811.s003.doc]

**Supporting Text S3. Segmental Order Parameters**

For the CG simulations, the second-rank order parameter for each bond in the lipid tails was defined as

$$P_{2}=\frac{1}{2}\left(3\left\langle\cos^{2}\theta\right\rangle-1\right)$$

where *θ* is the angle between the bond and the bilayer normal. P_2_ = 1 indicates parallel alignment of the bond with the bilayer normal, P_2_ = - 0.5 indicated an anti-parallel alignment, and P_2_ = 0 indicates a random orientation. Addition of 2.3% TO had no effect on P_2_, while 5.2% TO caused a slight decrease in P_2_ because the POPC acyl tails sandwiching the aggregate in the center of the membrane in the 4X5 and 4XMID5 simulations pointed towards the aggregate, resulting in lower values of *cosθ*. However, the packing of POPC molecules far away from the aggregate is mostly undisturbed.
